# Simulation of daily soft multifocal contact lenses using SimVis Gekko: from in-vitro and computational characterization to clinical validation

**DOI:** 10.1038/s41598-024-59178-1

**Published:** 2024-04-13

**Authors:** Eduardo Esteban-Ibañez, Diego Montagud-Martínez, Lucie Sawides, Amal Zaytouny, Alberto de Castro, Irene Sisó-Fuertes, Xoana Barcala, David P. Piñero, Walter D. Furlan, Carlos Dorronsoro, Enrique Gambra

**Affiliations:** 1 2EyesVision SL, Plaza de la Encina, 10, Nucleo 3, Planta 4ª, 28760 Tres Cantos, Madrid, Spain; 2 Institute of Optics ‘Daza de Valdés’, Spanish National Research Council, IO-CSIC, Madrid, Spain; 3 Centro de Tecnologías Físicas, Universitat Politècnica de València, Valencia, Spain; 4 Departamento de Óptica y Optometría y Ciencias de la Visión, Universitat de València, Valencia, Spain; 5 Departamento de Óptica, Farmacología y Anatomía, Universidad de Alicante, Alicante, Spain

**Keywords:** Optical metrology, Biophotonics

## Abstract

Multifocal contact lenses (MCLs) are one of the solutions to correct presbyopia, but their adoption is not widespread. To address this situation, visual simulators can be used to refine the adaptation process. This study aims to obtain accurate simulations for a visual simulator (SimVis Gekko; 2EyesVision) of daily soft MCL designs from four manufacturers. In-vitro characterization of these MCLs—several powers and additions- was obtained using NIMO TR-1504. From the averaged relative power profiles across powers, phase maps were reconstructed and the Through-Focus Visual Strehl metric was calculated for each MCL design. The SimVis Gekko simulation corresponding to each MCL design was obtained computationally and bench-validated. Finally, the MCL simulations were clinically validated involving presbyopic patients. The clinical validation results show a good agreement between the SimVis Gekko simulations and the real MCLs for through-focus visual acuity (TF-VA) curves and VA at three real distances. All MCL designs showed a partial correlation higher than 0.90 and a Root Mean Square Error below 0.07 logMAR between the TF-VA of simulations and Real MCLs across subjects. The validity of the simulation approach using SimVis Gekko and in-vitro measurements was confirmed in this study, opening the possibility to accelerate the adaptation of MCLs.

## Introduction

Presbyopia is commonly defined as the loss of capability to focus correctly, using the crystalline lens, at intermediate or near distances. This usually occurs at 45 years old and progresses with age to the point of losing the ability completely. This condition has been explained as a loss of crystalline lens elasticity by several authors^1,2^.

Due to the global increase in the aging population, the number of people affected by presbyopia is constantly growing, expecting to reach 2.1 billion worldwide in 2030^3^. Different correction strategies are available to compensate this condition and overcome its symptoms, using different platforms such as spectacles, intraocular lenses (IOLs), contact lenses (CLs) and corneal laser refractive surgery (PresbyLASIK) treatments. These strategies mostly differ in the optical design approach, which includes monofocal corrections, progressive lenses (mostly in spectacles), monovision strategies, bifocal lenses, trifocal lenses, extended depth of focus (EDOF) and monofocal enhanced corrections. The options are increasing exponentially, as new commercial solutions are continuously appearing in the market and different corrections can be applied in each eye (mix-and-match, blended vision…).

Despite the existence of bifocal and multifocal designs in CLs (MCLs), the percentage of CL users decreases when presbyopia is reached. This tendency was demonstrated in the study of Naroo et al.^4^, in which the percentage of CL users changed from 66 to 52% and 25% among the 40–44, 45–49 and 65–70 years age groups, respectively. Also in this study, only 25% of presbyopic CL users wore a multifocal or multifocal toric design, a very similar proportion (29%) to the one found by Morgan et al.^5^. Both facts can be explained by various reasons: ocular dryness and associated discomfort, unawareness of multifocal solutions, unexpected visual performance and time-consuming fitting process to obtain a success adaptation, leading to several visits to eye care practitioner. Some of these unexpected visual performance aspects can be related to photic phenomena (i.e. glare and dysphotopsias), contrast decrease or an insufficient near vision to perform routinary tasks^6^.

Visual simulators can help patients understand how their vision would be like with different MCL designs, allowing clinicians to recommend the most suitable option considering patient’s visual needs and expectations, thus improving the process. There have been a few attempts to produce commercial visual simulators based on different technologies (adaptive optics, projection of a real lens in a cuvette…), but most of them have not yet validated its ability to replicate commercial corrections. Moreover, these devices only offer monocular simulation, many of them displaying visual stimuli on an internal screen, and all of them are designed as tabletop devices.

The SimVis Gekko visual simulator (2EyesVision SL, Spain), based on temporal multiplexing using optotunable lenses, has already demonstrated its ability to accurately simulate multifocal IOL designs using data from the public-literature of visual quality metrics^7–9^. Moreover, the potential impact of SimVis technology for the CL market, exploiting its ability to simulate all kinds of multifocal corrections binocularly, has already been successfully demonstrated in a previous study performed in clinical settings using 1-Day Acuvue Moist MCLs^10^. SimVis Gekko offers key advantages that could enhance the fitting of MCLs including the ability to test different simulations in each eye independently. SimVis Gekko also allows a see-through vision of natural stimuli at different distances, providing a broad field of view (20°) with a wearable device.

Information on the power profile of the MCLs is needed to achieve correct simulations. Potentially, this information can be obtained from CL manufacturers, from public data about the MCLs designs or metrology measurements. MCLs can be characterized by measuring their power profile across the surface lens using dedicated devices for this purpose. To the best of our knowledge, three commercial devices have been used to measure MCL power profiles in different studies: NIMO TR-1504 (Lambda X, Belgium)^11^, SHSOphthalmic (Optocraft, Germany)^12^ and Phase Focus Lens Profiler (Phase Focus Ltd, UK)^13^. Nevertheless, the measurements obtained by these instruments are not free of drawbacks such as repeatability problems, calibration processes required and accuracy uncertainties with the devices compared to data provided by manufacturers. These discrepancies may be attributed to variations in the manufacturing process that diverge from the theoretical design^14^, assuming a closer approximation to the final lens design which can also vary with the nominal power^11^. Moreover, most of the MCLs measured in the literature are monthly replacement designs, while the market trends towards a greater use of daily replacement and silicone hydrogel materials^15^.

Our main objective in this study was to obtain accurate SimVis Gekko simulations of different daily commercial soft MCL designs from four manufacturers. For this goal, first, we obtained an in-vitro and computational characterization of these MCLs using NIMO TR-1504 and a dedicated algorithm, and then we clinically validated the SimVis Gekko simulations, obtained from these characterizations, in a small group of patients in a pilot study.

Finally, the obtention of these simulations could provide eye care practitioners with a powerful tool to non-invasively demonstrate the benefits and disadvantages of each MCL design. It allows patients to actively participate in the fitting process, test multiple MCL designs instantly in a single session and speed up the overall process.

## Methods

### MCL characterization

#### MCLs measured: four different families

Four families of daily commercial soft MCLs from different manufacturers were studied: MyDay (CooperVision, USA), 1-Day Acuvue Moist (Johnson & Johnson, USA), Dailies Total1 (Alcon, USA) and Biotrue ONEday (Bausch + Lomb, USA). The different parameters of the lenses studied can be seen in Table 1. MCLs with distance powers of − 4.00 D, − 2.00 D, 0.00 D, + 2.00 D and + 4.00 D and additions Low, Mid and High were measured. Powers of -4.00 D and + 4.00 D were also measured only in MyDay and Dailies Total1, as a comprehensive characterization, because there is no literature data for their power profiles, as far as we know. The Biotrue ONEday MCL had only Low and High additions available.

**Table 1:** Nominal parameters of the four daily commercial soft MCL families used in this study.

Parameter	MyDay	1-Day Acuvue Moist	Dailies Total1	Biotrue ONEday
Manufacturer	CooperVision	Johnson & Johnson	Alcon	Bausch + Lomb
Add	Low Mid High	Low Mid High	Low Mid High	Low High
Distance power (D)	− 4.00 − 2.00 + 2.00 + 4.00	− 2.00 0.00 + 2.00	− 4.00 − 2.00 0.00 + 2.00 + 4.00	− 2.00 0.00 + 2.00
Material	Stenfilcon A	Etafilcon A	Delefilcon A	Nesofilcon A
Refractive index (546 nm)	1.40	1.40	1.42	1.37
Lens design	Aspheric	Aspheric	Aspheric	Aspheric
Water content (%)	54	58	33	78
Base curve (mm)	8.4	8.4	8.5	8.6
Diameter (mm)	14.2	14.3	14.1	14.2
Central thickness (mm) @-3.00 D	0.08	0.084	0.09	0.1

#### NIMO TR-1504 device

The MCLs were measured with the instrument NIMO TR-1504 (Lambda-X, Belgium), an optical mapping device which allows measuring the power profile of CLs^16^. It is based on the patented quantitative deflectometry technique, combining the Schlieren principle with a phase shift method. It uses a light source of 546 nm to measure beam deflections and obtain power maps. Its accuracy and repeatability are 0.05 D for spherical soft CLs powers and between 0.04 and 0.2 D for MCLs^16,17^.

#### MCLs power profile measurement process

First, a calibration of the device was performed, following the method recommended by the manufacturer using standard calibration lenses. Then, each MCL was taken out of the blister pack and placed in a cuvette with saline solution at room temperature. The parameters of each MCL unit (see Table 1) were introduced into the device's software and the MCL was centered using the device's camera. The power profile was measured across a 6 mm diameter optical zone: from MCL center (0 mm) to 3 mm of semi-diameter. To check the repeatability, the measurement process -removing and re-introducing the MCL in the cuvette- was repeated 2 more times*.* The power profiles, obtained for each MCL addition and distance power, were processed with a moving average filter with a window size of 5. The results of multiple repetitions were averaged to calculate the final power profiles.

#### Computational process: obtaining phase map and theoretical through-focus visual Strehl

The average power profile of each MCL design for all distance powers was used to compute the Through-Focus Visual Strehl (TF-VS) for 5 different diameters (from 3 to 5 mm in a 0.50 mm step) using a custom algorithm in Matlab (MathWorks, USA). Note that through these TF-VS, only the pure multifocal pattern of the MCLs is considered. Essentially, the 2-dimensional wavefront aberration map (phase map) was calculated point-by-point so that its curvature resulted in the power profile measured for each MCL design, and Fourier Optics was used to calculate the modulation transfer function (MTF). TF-VS is an optical quality metric that quantifies the volume under the MTF at all spatial frequencies weighted by the human contrast sensitivity function and has been recognized as a good predictor of visual acuity^18,19^. Furthermore, it has been validated as an input metric for SimVis Gekko simulations^7,20,21^. This metric was then evaluated in a range from − 2.00 D to + 4.00 D in 0.05 D steps. Phase maps were obtained for each MCL design (for each family and addition) and the TF-VS was calculated for each optical diameter.

### Validation of SimVis Gekko simulations

#### Computational calculation of SimVis simulations

From the TF-VS of each lens design, a set of SimVis temporal coefficients were calculated based on the equation described by Akondi et al.^20,21^. The set of time coefficients describes the lens simulation with SimVis Gekko, indicating how long the tunable lenses of the instrument must stay in each focus (in 0.1 D steps) under temporal multiplexing to replicate the desired multifocal element. Mathematically, the temporal coefficients stand for the weighting factors of a series of monofocal point spread functions (PSFs) for different optical powers (additions), tuned to match the TF-VS of the MCL design. The number of temporal coefficients varies according to the lens design.

#### On-bench TF-VS validation

Each MCL SimVis Gekko simulation was experimentally evaluated on-bench with a high-speed focimeter^21,22^ provided with a camera working at 3823-fps (IL5S; Fastec Imaging, USA) and an optotunable lens (Optotune, Switzerland, EL-3-10). The measurements were carried out in two different sessions with a room temperature fixed at 26 °C. The TF-VS ratio was computed from the experimental measurements obtained with the high-speed focimeter for the SimVis lens simulation, using specific custom Matlab programs. The time spent at each optical power is calculated (in 0.1 D steps), and the differences with respect to the theoretical TF-VS were assessed in terms of Root Mean Square Error (RMSE). The on-bench validation was successfully achieved if the following quality terms were reached: differences lower than 0.20 D shift for the location of the TF-VS peaks and a RMSE less than 0.05 between theoretical and experimental TF-VS in the dioptric range of interest: − 1.00 D to + 4.00 D.

#### SimVis Gekko visual simulator

SimVis Gekko^23,24^ is a see-through and head mounted clinical visual simulator with the capability to mimic binocularly any multifocal design behavior, such as IOLs^7–9^ (including patients with mild cataract opacification^25^), CLs^10,26^ and presbyLASIK patterns^27^. SimVis Gekko simulates these multifocal elements working under temporal multiplexing using optotunable lenses^20^, generating fast and periodic optical power changes at a speed greater than the defocus flicker fusion of the human eye. A SimVis Gekko™ device (v.0.8, 2022), with a pupil entrance of 3 mm, was used in the pilot study to clinically validate six MCL design simulations from three manufacturers in volunteer subjects.

Subjects’ refraction was corrected using trial lenses placed in the dedicated external trial lens holders attached to SimVis Gekko. Each patient’s pupil was centered vertically and horizontally with respect to the optical axis of the respective SimVis Gekko optical channel by using mechanical adjustments and a pair of color LEDs to guide the alignment process, allowing centration on the pupillary reflex and considering then the interpupillary distance.

#### Subjects

Eight presbyopic subjects (2 males and 6 females) participated in the pilot study (see Table 2). The protocol conducted in this study was approved by the Consejo Superior de Investigaciones Cientificas (CSIC) Ethics Committee and was performed in accordance with the guidelines of the Declaration of Helsinki. All subjects received a thoughtful explanation of the purpose of the study and signed an informed consent form.

**Table 2:** Subjects’ data related to age, gender, and ocular parameters: evaluated eye, refractive error (Sphere, Cylinder and Axis), near addition and mean pupil diameter (matched simulation pupil size using SimVis Gekko).

Subject	Age (y)	Gender	Evaluated Eye	Refractive Error (D, D, °)	Addition (D)	Pupil diameter (SV simulation) (mm)
S#1	45	F	OS	− 2.75–0.50 × 160	+ 0.75	3.90 (4.00)
S#2	45	F	OD	+ 2.50	+ 1.00	4.40 (4.50)
S#3	48	M	OS	− 4.00–0.50 × 100	+ 0.75	2.80 (3.00)
S#4	47	F	OS	+ 2.00–0.50 × 180	+ 1.25	2.90 (3.00)
S#5	62	F	OS	+ 2.75–0.50 × 100	+ 2.25	3.10 (3.00)
S#6	61	F	OS	+ 1.75–0.50 × 110	+ 2.00	3.50 (3.50)
S#7	53	M	OD	− 3.25–0.25 × 165	+ 1.75	3.50 (3.50)
S#8	50	F	OS	− 1.50–0.50 × 90	+ 2.00	3.80 (4.00)

Subjects were distributed in two different groups according to their near addition: young presbyopes (46.3 ± 1.3 years) including subjects with a near add from + 0.75 to + 1.25 D and old presbyopes (56.5 ± 5.9 years) including subjects with a near add from + 1.75 to + 2.50 D. Each addition group had 2 hyperopes and 2 myopes with a spherical refractive error equal to or higher than 1.50 D in absolute terms. The exclusion criteria were astigmatism higher than 0.75 D, contraindications for CL use, previous ocular surgery and/or pathology.

#### Clinical validation measurements (pilot study)

Prior to clinical validation, a complete optometric examination (anamnesis, subjective refraction, near addition quantification, ocular dominance and slit lamp examination) was carried out for all subjects to obtain the required information and ensure that all of them met the inclusion criteria. Clinical measurements consisted of monocular Through-Focus Visual Acuity (TF-VA) curves with trial lenses ranging from + 1.00 to − 4.00 D (in 0.50 D steps). Monocular visual acuity (VA) was also measured at real distances (4 m, 0.66 m and 0.40 m). These measurements were performed for SimVis Gekko simulated MCL and for Real MCL Biotrue ONEday, MyDay and Dailies Total1 designs considering the subject’s addition. 1-Day Acuvue Moist was not included in the pilot study as this MCL design had already been clinically validated for all additions using SimVis Gekko in Barcala et al.^10^. We evaluated only the dominant eye in subjects fitted with Low addition MCLs and the non-dominant eye in subjects fitted with High addition MCLs, following the recommendations of the fitting guide of each brand.

The VA charts of ETDRS optotypes were displayed into Optonet Vision Unit (Optonet Ltd, United Kingdom) in a calibrated screen: 48.5-inch screen (49UH850V, LG) for 4 m distance and iPad Pro 12.9-inch for 0.66 m and 0.40 m distances. VA measurements were performed in a dark room where only the display with the optotypes was illuminated. The luminance of the display was 200 cd/m^2^, measured with ColorCal Colorimeter (Cambridge Research Systems, UK), as recommended by ISO 10938:2016 ^28^.

The Plusoptix Power Refractor II (Plusoptix, Germany) was exclusively used to measure the patients’ pupil size three times. The light conditions during these pupil size measurements were the same as those applied in the clinical evaluation of real MCLs visual performance. Since MCL performance depends on the pupil size, we selected the mean pupil size for each subject to determine the appropriate MCL SimVis simulations. The developed simulations were applied using the closest value to the real pupil size, rounded to the nearest 0.50 mm step. If the subject had a sphero-cylindrical refraction (obtained from subjective refraction exam), it was corrected with trial lenses with SimVis Gekko and the composition of spherical error and tear film meniscus with the Real MCL, since astigmatism was less than 0.75 D in all subjects.

For real MCL evaluation, we waited 10 min for a correct CL settlement, before evaluating the fitting quality according to the CLEAR clinical guideline^29^. A randomized criteria was established to choose the evaluation order for the lens tested (SimVis simulation or Real MCL) and brands examined.

#### Data analysis

The Depth of Focus (DoF), estimated as the range of values with a Strehl ratio higher than 0.12 (VS > 0.12)^30,31^, was calculated for the theoretical TF-VS for 3 mm, 4 mm and 5 mm of diameter, in each SimVis Gekko simulation obtained computationally, to analyze behavioral changes between different diameters and additions.

Two different metrics were used to compare the SimVis Gekko simulations with the Real MCLs in TF-VA terms using a dedicated Matlab script: partial correlation (r_xy,z_)^32^, where the SimVis Gekko simulation TF-VA was defined as *x*, the Real MCL TF-VA as *y*, and the defocus value as z; and RMSE between the SimVis Gekko simulation TF-VA and the Real MCL TF-VA.

## Results

### MCLs characterization

**Figure 1:**
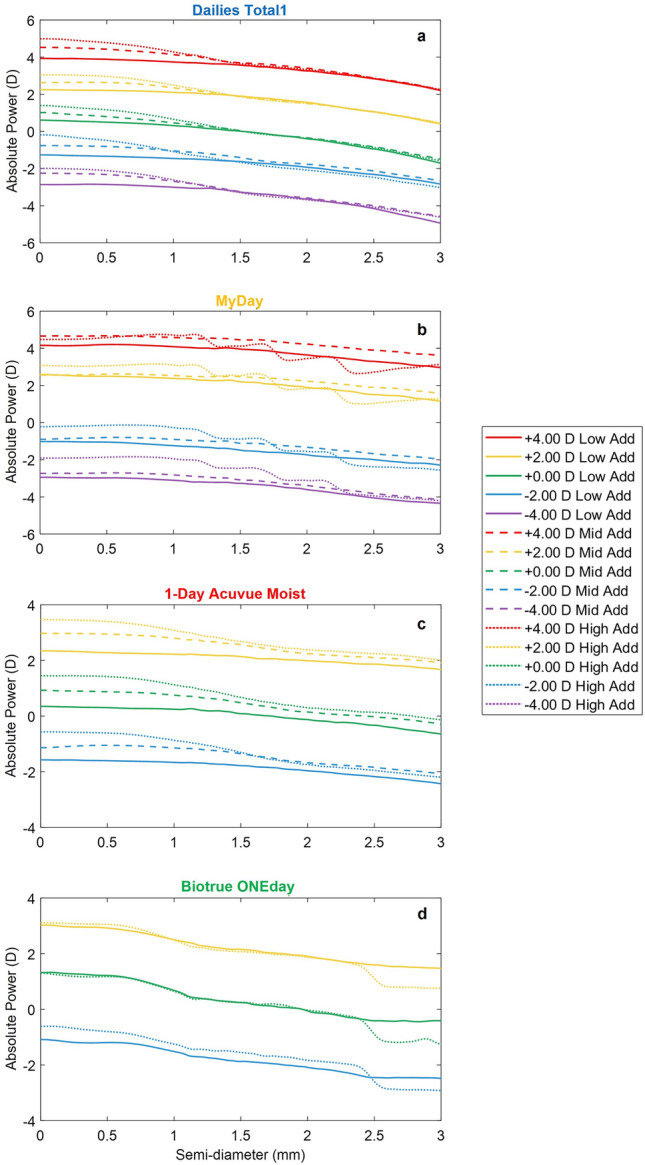
Absolute power profiles measured with NIMO TR-1504 across 3 mm of semi-diameter for each MCL family: (**a**) Dailies Total1, (**b**) MyDay, (**c**) 1-Day Acuvue Moist, and (**d**) Biotrue ONEday. The different additions are represented for each family with solid lines (Low Addition), dashed lines (Mid Addition) and dotted lines (High Addition) with different colors according to the labeled distance power: red (+ 4.00 D), orange (+ 2.00 D), green (0.00 D), blue ( − 2.00 D) and purple (− 4.00 D).

Figure 1 shows the absolute power profiles measured for each lens family, addition and distance power of MCLs (a total of 42 power profiles and 126 measurements) as a function of the semi-diameter up to a maximum of 3 mm. The average of the three repetitions is shown in Fig. 1; the standard deviation was less than 0.10 D along all the semi-diameters for each MCL design.

The two typical MCL designs followed by most of manufacturers are shown in Fig. 1: Dailies Total1 (Fig. 1a), MyDay Low and Mid Addition (Fig. 1b) and 1-Day Acuvue Moist (Fig. 1c) with an aspheric center-near design with a power decrease towards the MCL periphery, while MyDay High Addition (Fig. 1b) and Biotrue ONEday (Fig. 1d) have a concentric center near design with different number of steps between designs, four and three, respectively.

**Figure 2:**
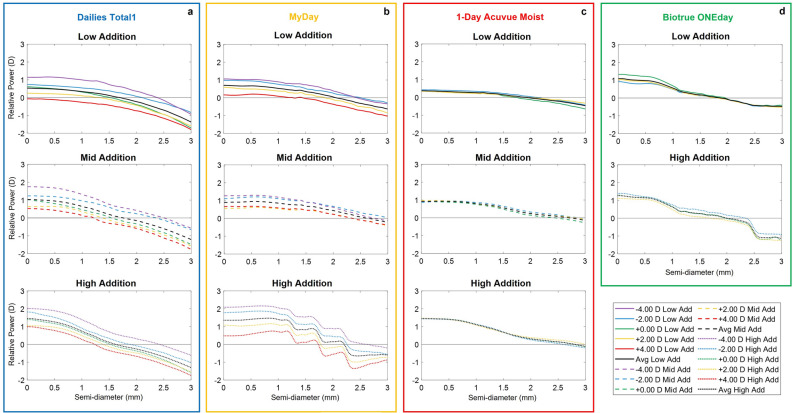
Relative power profiles obtained through NIMO TR-1504 measurements across 3 mm of semi-diameter for each MCL family: (**a**) Dailies Total1, (**b**) MyDay, (**c**) 1-Day Acuvue Moist, and (**d**) Biotrue ONEday. The different additions are represented for each family with solid lines (Low Add), dashed lines (Mid Add) and dotted lines (High Addition) with different colors according to the labeled distance power: red (+ 4.00 D), orange (+ 2.00 D), green (0.00 D), blue (− 2.00 D) and purple (− 4.00 D). The average (Avg) relative power profile across all distance powers is represented in black solid line for each lens design.

For a comprehensive analysis and better comparison between MCL designs, relative power profiles (subtracting the distance power, in order to reveal the multifocal contribution) are presented in Fig. 2, including the average across distance powers for each MCL design (in black lines).

While 1-Day Acuvue Moist and Biotrue ONEday families show a very similar power profile across distance powers, Dailies Total1 and MyDay exhibit differences in the relative power profiles across distance powers. The latter families present relative power profiles with more power for negative distance powers and less power for positive distance powers, with a maximum difference of 1.61 D between the − 4.00 D and the + 4.00 D lenses for the MyDay High Addition.

Considering the average power profiles, addition differences across MCL families can be observed. The lowest and highest addition are found for 1-Day Acuvue Moist (0.37 D) and Biotrue ONEday (1.09 D) in low addition designs, for MyDay (0.90 D) and Dailies Total1 (1.03 D) in mid addition designs, and for Biotrue ONEday (1.27 D) and Dailies Total1 (1.45 D) in high addition designs, respectively.

**Figure 3:**
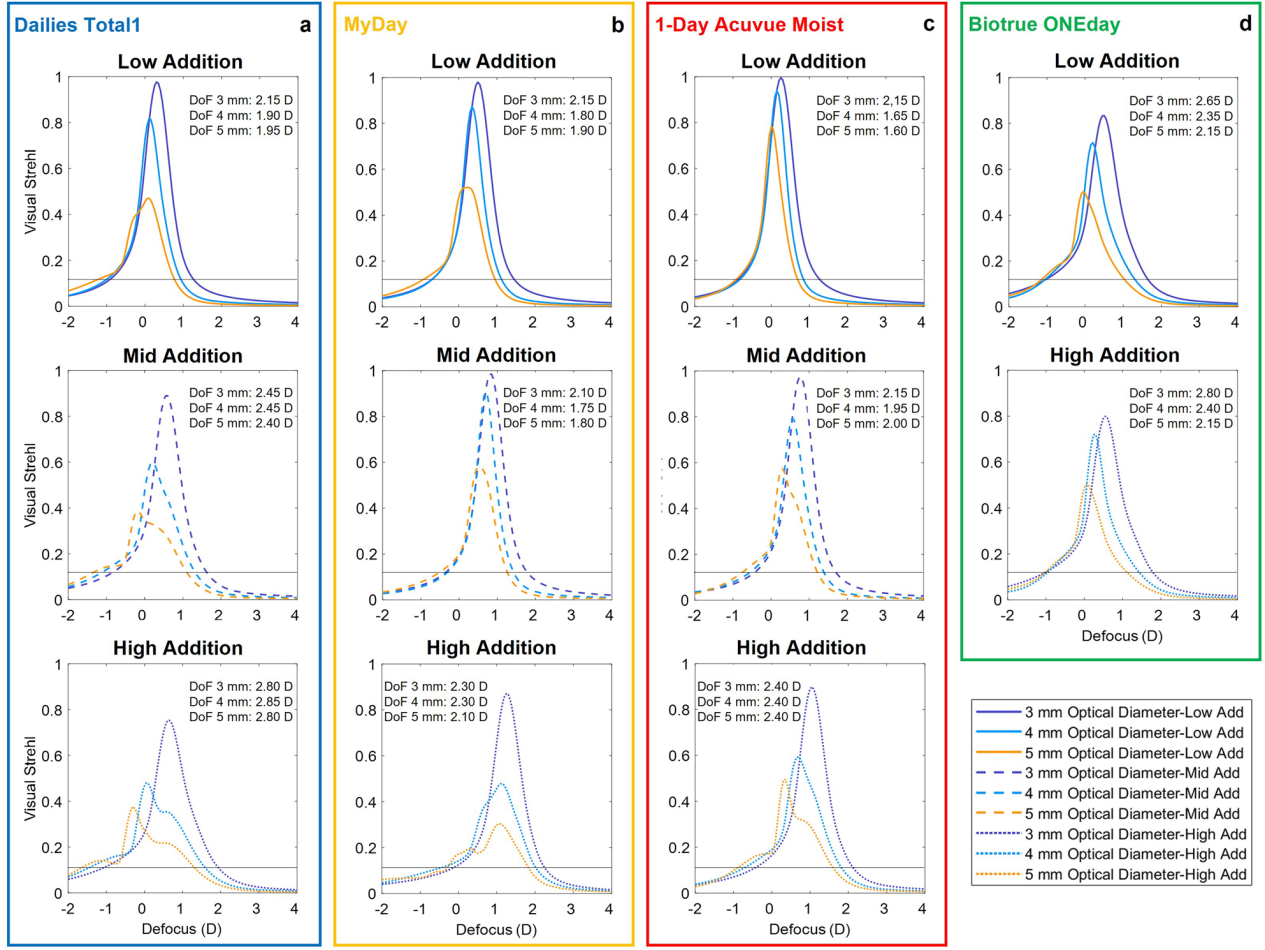
Theoretical TF-VS computationally calculated to simulate MCLs with SimVis Gekko for each family: (**a**) Dailies Total1, (**b**) MyDay, (**c**) 1-Day Acuvue Moist, and (**d**) Biotrue ONEday. The TF-VS for each family is represented with solid, dashed and dotted lines for low, mid and high additions, respectively, with different colors for optical diameter zone of 3 mm (purple), 4 mm (blue) and 5 mm (yellow). The DoF estimated as the range where VS > 0.12 is shown for each MCL design for the three optical diameters.

The theoretical TF-VS, calculated using the average power profiles across distance powers for each MCL design, are shown in Fig. 3 for 3 mm, 4 mm and 5 mm optical diameters. There is a tendency to vary from a more monofocal performance, provided by near addition, for 3 mm of optical diameter, towards an increase in the DoF as the optical diameter increases, shifting the main peak towards the distance power (except for MyDay High Addition).

If we analyze the TF-VS for different additions, we can observe an increase in the DoF (VS > 0.12), as the addition increases. The DoF, for the 4 mm optical diameter, between low and high addition changes from 1.90 to 2.85 D for Dailies Total1, 1.80 D to 2.30 D for MyDay, 1.65 D to 2.40 D for 1-Day Acuvue Moist and 2.35 D to 2.40 D for Biotrue ONEday.

### Clinical validation of the SimVis Gekko simulations

**Figure 4:**
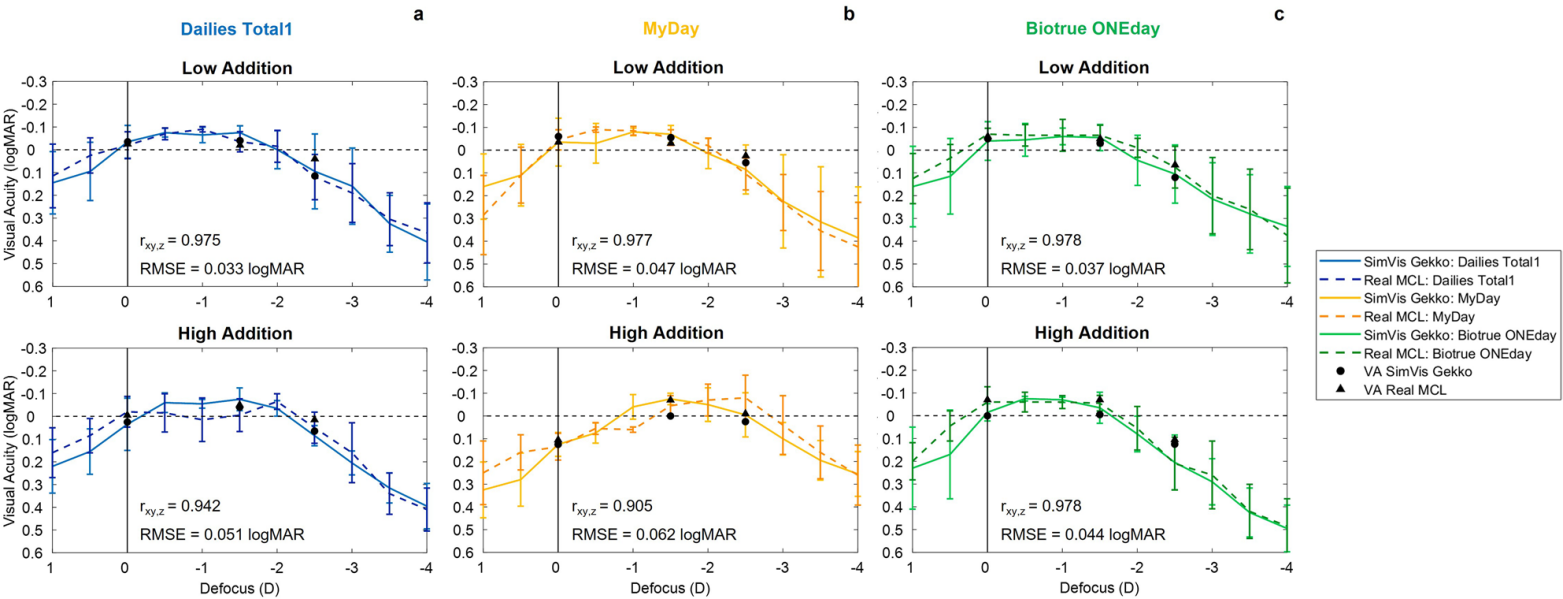
Clinical validation of simulated MCLs using SimVis Gekko for each addition group (n = 4). TF-VA curves and real distances VA measurements with both the three families of real MCLs and the respective MCLs simulations using SimVis Gekko: (**a**) Dailies Total1 (blue color) (**b**) MyDay (yellow color) and (**c**) Biotrue ONEday (green color); for low (upper row) and high addition (bottom row). The TF-VA (with standard deviation in each 0.50 D step) and real distances VA are represented by dashed lines and black triangles for Real MCLs and solid lines and black circles for the simulated MCLs, respectively. The degree of correlation between methods for each MCL design is provided in each graph by partial correlation (r_xy,z_) and RMSE metrics.

**Figure 5:**
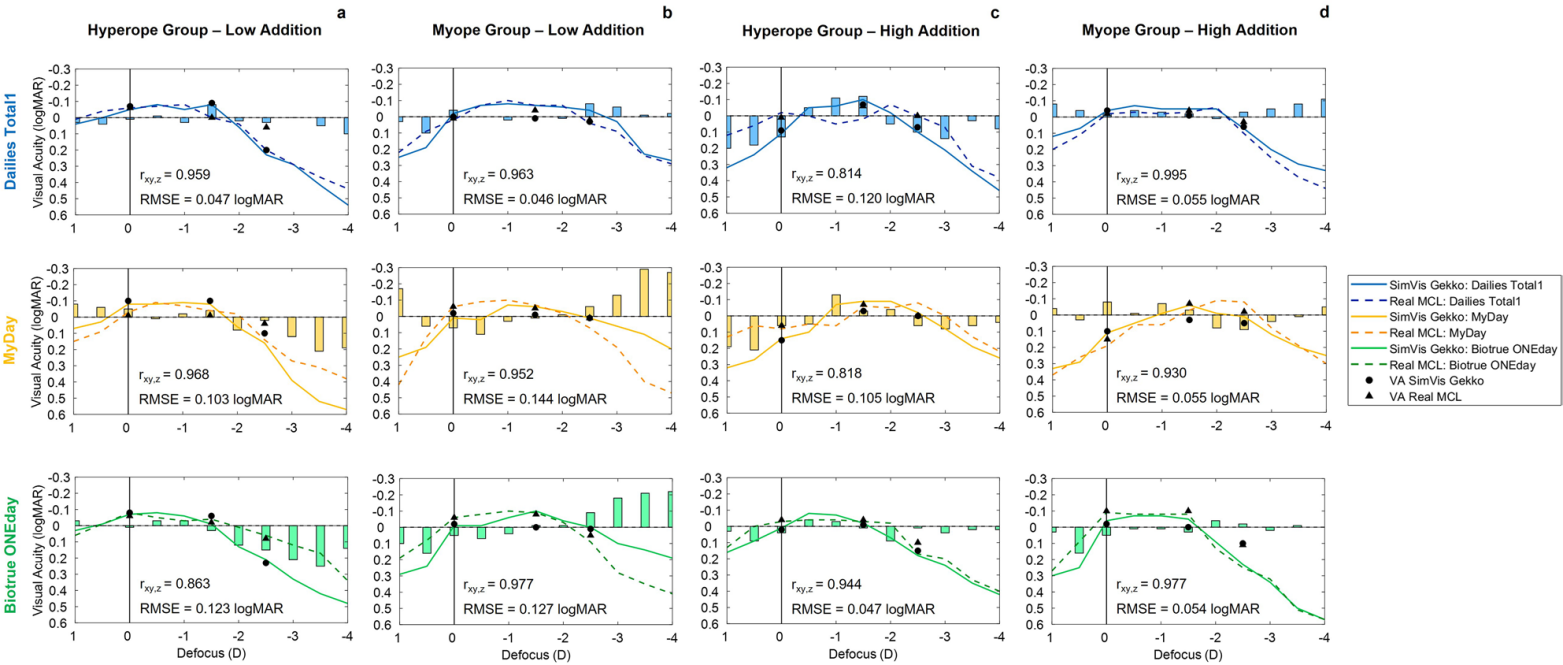
Clinical validation of simulated MCLs using SimVis Gekko for each subgroup: refractive error and addition. TF-VA curves and real distances VA measurements with both the real MCLs and the respective MCLs simulations using SimVis Gekko for different sample groups (n = 2), considering addition and also the sign of the refractive error: (**a**) Hyperope-Low Addition, (**b**) Myope-Low Addition, (**c**) Hyperope-High Addition and (**d**) Myope-High Addition, with Dailies Total1 (blue color; upper row), MyDay (yellow color; middle row) and Biotrue ONEday (green color; bottom row). The TF-VA and real distances VA are represented by dashed lines/black triangles and solid lines/black circles for Real MCLs and simulated MCLs, respectively. Vertical bars represent the TF-VA differences between simulated MCLs and Real MCLs in each 0.50 D step. The degree of correlation between methods for each MCL design is provided in each graph by partial correlation (r_xy,z_) and RMSE metrics.

The measurements performed in the clinical validation with the SimVis Gekko simulations of MCLs are shown in Figs. 4 and 5. TF-VA and VA are presented comparing the results obtained with SimVis Gekko simulations (solid lines and black circle markers) and with real MCLs (dashed lines and black triangle markers) for low and high additions. The mean pupil size across all patients when wearing real MCLs for low and high addition was 3.44 ± 0.78 mm and 3.47 ± 0.29 mm, respectively, while the mean pupil size used for SimVis simulations was 3.57 ± 0.75 mm and 3.48 ± 0.41 mm for the same additions.

The comparative metrics reveal a range of partial correlation (r_xy,z_) and RMSE between 0.905 and 0.978 and 0.033 logMAR and 0.062 logMAR for all lens designs studied. Mean differences of VA in real distances between Real MCL and SimVis Gekko simulations (Real MCL VA—SimVis VA) were calculated across all distances for each manufacturer, being 0.023 ± 0.036 logMAR for Dailies Total1, 0.018 ± 0.037 logMAR for MyDay and 0.04 ± 0.026 logMAR for Biotrue ONEday. In addition, mean values of 0.012 ± 0.036 logMAR in low addition and 0.041 ± 0.023 logMAR in high addition were obtained. The overall mean differences, considering the three distances evaluated, were 0.016 ± 0.033 logMAR, 0.020 ± 0.041 logMAR and 0.040 ± 0.020 logMAR for 4 m, 0.66 m and 0.40 m, respectively.

In order to carry out a comprehensive analysis between SimVis Gekko simulations and Real MCL, comparative results for each refractive error group and addition are shown in Fig. 5, including bars that report the VA differences between methods in each TF-VA step. Partial correlation and RMSE metrics are also calculated: for the low addition design in the hyperope group and across all manufacturers, r_xy,z_ and RMSE are 0.929 ± 0.058 and 0.084 ± 0.039 logMAR, while the results are 0.964 ± 0.013 and 0.094 ± 0.052 logMAR in the myope group for the same addition. Otherwise, the partial correlations and RMSE for high addition in the hyperope group and myope group are 0.857 ± 0.74, 0.967 ± 0.034 and 0.084 ± 0.039 logMAR, 0.055 ± 0.001 logMAR across manufacturers, respectively.

## Discussion

An in-vitro and computational characterization of daily soft MCLs from four different manufacturers is reported, using the NIMO device and a dedicated algorithm based on the in-vitro measurements, respectively. These characterizations were employed as an independent input source (not provided by any manufacturer) to obtain the corresponding SimVis Gekko simulations. Finally, the MCL simulations have been clinically validated in a pilot study with volunteer subjects having different refractive errors and additions.

The absolute power profiles (see Fig. 1) show a consistent behavior with respect to distance powers and additions, except for Biotrue ONEday which had similar designs for low and high additions up to 2.5 mm of semi-diameter. MyDay results reveal different designs for low/mid (continuous aspheric design) and high additions (concentric rings).

The relative power profiles (see Fig. 2) highlight differences with the distance power for Dailies Total1 and MyDay families, while 1-Day Acuvue Moist and Biotrue ONEday families present equivalent profiles across distance powers, coinciding with the results obtained by Kim et al.^11^ using NIMO TR-1504. These differences across distance powers have been previously reported in other studies^11,33^, where a higher value of positive spherical aberration have been found for positive distance power, while a higher amount of negative spherical aberration have been found for negative distance powers^11^. This phenomenon might be explained based on the strategy followed by some manufacturers of considering the eye’s inherent spherical aberration and compensating it with the amount introduced by their MCLs to improve visual performance^34,35^. SimVis Gekko only simulates the addition profile, not the refractive correction component nor the interaction between the lens and the eye spherical aberration. Therefore, we decided that averaging across all distance powers, considering the symmetry between positive and negative measured distance powers, would be the fairest estimation to represent the addition for each lens design.

The theoretical TF-VS, calculated using the average relative power profiles, shows two distinct behaviors: (1) nearly monofocal with a low DoF (low/mid addition), addressed to presbyopes with residual accommodative amplitude, and (2) a more multifocal behavior, increasing with pupil size, for older presbyopes. For high addition designs, the CLs behave as multifocal with most energy devoted to the far focus for 5 mm pupil diameters, except MyDay which prioritizes near vision (see Fig. 3). These differences between designs for the same addition label, especially high, are consistent with the different strategies followed by each manufacturer in their fitting guides, as they can opt for a symmetric addition in both eyes (Dailies Total1 and Biotrue ONEday) or a different addition depending on whether it is dominant or non-dominant eye (1-Day Acuvue Moist and MyDay).

The clinical validation shows that the SimVis Gekko simulations for different additions and lens families have a good agreement with real MCLs when measuring TF-VA curves and VA at real distances (see Fig. 4). As the TF-VS changes significantly with pupil size, simulations for every MCL design were obtained and used in the clinical validation between 3 and 5 mm (in 0.5 mm steps). The good agreement observed between the results obtained with the MCLs and SimVis Gekko simulations suggest that considering a sampling of the lens design simulated with SimVis Gekko every 0.5 mm pupil size provides an accurate simulation of the lens design. The low addition designs had slightly better agreement than high additions designs in both partial correlation (r_xy,z_) and RMSE in the TF-VA curve, this small difference being probably due to higher variability when using lenses with a significant multifocal behavior. The differences in real distance VA between methods, although small, showed better VA with the real MCLs, for each manufacturer and addition across the three tested distances, with a maximum difference of 2 logMAR letters for MyDay and high addition respectively. Similarly, the largest difference between methods considering the distance, across all designs and manufacturers, was observed at 0.40 m, also with a value of two letters. Although the tendency seen was VA is better with real MCLs, the total of these differences was within the variability of repeated VA measurements: 0.04 ± 0.06 logMAR^36^.

Moreover, a comparison between groups of subjects with the same addition and refractive error sign (n = 2) was conducted to assess the impact of relative profile differences on clinical simulations (see Fig. 5). Despite finding good correspondence between simulations and real MCLs results, better partial correlation and RMSE were observed for the myopic group with both low and high additions, with larger differences seen in the case of high additions. Among the hyperopic designs, there were three of them with higher differences between methods with a r_xy,z_ below 0.90 and RMSE beyond 0.10 logMAR: Biotrue ONEday Low Addition, MyDay High Addition and Dailies Total1 High Addition. This fact can be explained by the differences in the amount of spherical aberration between designs for hyperopes or myopes and how this spherical aberration interacts with the spherical aberration of the eye. However, this effect cannot be completely modeled with the SimVis Gekko, where we have used the average relative power profile to obtain the simulation of MCLs. Otherwise, the residual accommodation amplitude could have an influence in the spherical aberration value according to the refractive error group, since these differences between refractive groups occur mainly in young presbyopes, as in Biotrue ONEday family while it does not happen for the high addition design of the same lens family, despite having almost an identical power profile. Furthermore, it is crucial to consider the potential impact of variability within the small subject groups as a limitation of the pilot study that could affect the pupil size, amount of refractive error or even fatigue doing the measurements. In any case, it has to be noticed that these behaviors, although systematic, do not occur in the region of the defocus range where the SimVis Gekko simulation is expected to be really accurate, but in the two extremes of the defocus curve range (above + 0.00 D and below − 2.50 D), where it does not have such an impact on visual performance and the defocus is higher increasing the variability in VA results^37^.

In summary, the designs of four daily commercial soft MCLs from different manufacturers and different additions have been characterized using a commercial device (NIMO TR-1504) and the results have been used to produce a visual simulation of the corrections with SimVis Gekko. Clinical comparison between the visual acuity with the simulation and with the real MCL on eye for hyperopes and myopes with different additions were conducted. The good agreement in all the reported cases—even when considering separately groups with different refractive error, lens design and addition—confirms the validity of the simulation approach. SimVis Gekko simulations capture with a high degree of accuracy the multifocal performance of the lens, while there is still some room for fine tuning by calculating dedicated simulations for each refractive group.

After validation of SimVis Gekko's accuracy in simulating the performance of daily commercial soft MCLs, a study could be conducted to assess its potential use in clinical practice. This would allow for quick and accurate testing of different lens designs without the need to fit multiple MCL units. Furthermore, incorporating other clinical measurements using SimVis Gekko, such as contrast sensitivity or photic phenomena analysis, would provide a higher qualitative comprehension of the correlation with real MCLs.

In the future, a project with a larger sample size using these simulations could be carried out to replicate or even modify fitting guides of each manufacturer based on addition in order to streamline the process, save chair-time and enable testing of various designs in a single session.

## Data Availability

The datasets generated and analyzed during the current study are not publicly available at this time but may be obtained from the corresponding author on reasonable request.
